# Adverse childhood experiences among black sexually minoritized men and Black transgender women in Chicago

**DOI:** 10.1186/s12939-024-02168-6

**Published:** 2024-04-16

**Authors:** Christoffer Dharma, Katherine M. Keyes, Kara E. Rudolph, Cho-Hee Shrader, Yen-Tyng Chen, John Schneider, Dustin T. Duncan

**Affiliations:** 1 Department of Epidemiology, Mailman School of Public Health, Columbia University, 722 168Th St, New York, NY 10032 USA; 2 Dalla Lana School of Public Health, University of Toronto, Toronto, ON Canada; 3 Department of Public Health, William Paterson University of New Jersey, Wayne, NJ USA; 4 Department of Medicine, University of Chicago School of Medicine, Chicago, IL USA

## Abstract

**Background:**

Adverse childhood experiences (ACE) are important predictors of mental health outcomes in adulthood. However, commonly used ACE measures such as the Behavioural Risk Factor Surveillance System (BRFSS) have not been validated among Black sexually minoritized men (SMM) nor transgender women (TW), whom are known to have higher rates of ACE and poorer mental health outcomes. Assessing the psychometric properties of the measure is important for health equity research, as measurements that are not valid for some populations will render uninterpretable results.

**Methods:**

Data are drawn from the Neighborhoods and Networks (N2) study, a longitudinal cohort of Black SMM and TW living in Southern Chicago. We conducted confirmatory factor analysis, correlation analysis and a two-parameter Item Response Theory (IRT) on the BRFSS ACE measure, an 11-item measure with 8 domains of ACE.

**Results:**

One hundred forty seven participants (85% cisgender male) completed the BRFSS ACE measurement in the N2 study with age ranges from 16–34. The cohort were from a low socioeconomic background: about 40% of the cohort were housing insecure and made than $10,000 or less annually. They also have a high number of ACEs; 34% had endorsed 4 or more ACE domains. The three-factor structure fit the BRFSS ACE measure best; the measurement consisted of three subscales: of “Household Dysfunction”, “Emotional / Physical”, and “Sexual Abuse” (CFI = 0.975, TLI = 0.967, and RMSEA = 0.051). When the 8 domains of ACE were summed to one score, the total score was is correlated with depressive symptoms and anxiety scores, establishing concurrent validity. Item Response Theory model indicated that the “parental separation” domain had a low discrimination (slope) parameter, suggesting that this domain does not distinguish well between those with and without high ACE.

**Conclusions:**

The BRFFS ACE measure had adequate reliability, a well-replicated structure and some moderate evidence of concurrent validity among Black SMM and TW. The parental separation domain does not discriminate between those with high and low ACE experiences in this population. With changing population demographics and trends in marriage, further examination of this item beyond the current study is warranted to improve health equity research for all.

**Supplementary Information:**

The online version contains supplementary material available at 10.1186/s12939-024-02168-6.

## Background

Adverse childhood experiences (ACE) are important predictors of poor mental health outcomes such as depressive symptoms, anxiety, and mental distress in adulthood [1, 2]. ACE scores are higher in vulnerable groups such as Sexual and Gender Minorities (SGM) (e.g., Sexually Minoritized Men (SMM) and transgender individuals) compared to heterosexuals and has been hypothesized to mediate the relationship between sexual orientation and mental health [1–4]. It is also known that Black individuals who grew up in underserved communities with higher crime rates and poverty are more likely to endorse ACE items compared to the general populations [5]. Fewer studies have examined ACE within the intersection of Black SMMs however, which is expected to have even higher ACE relative to other intersectional groups.

Many studies commonly utilize the Behavioural Risk Factor Surveillance System (BRFSS) ACE measure, which has been shown to be invariant (i.e. having the same structure and properties) across different groups, including by sexual orientation and ethnicity [1, 2, 6–8]. The measure is commonly used to identify health inequities due to ACE [2, 7]. However, it has not been evaluated specifically within Black SMM, nor among any transgender women (TW) [8, 9]. There are reasons to believe existing ACE measurements might be inadequate for Black SMMs and may not capture community context, where general racial inequities in ACE exist due to higher incarceration, higher rates of parental absence and separation, other institutional disparities [5, 10]. It is possible that due to the higher prevalence of these ACE domains (i.e., parental separation, incarceration) within the population, the existing ACE measure may not be able to distinguish between Black SMM who were experiencing high and low ACE. If the underlying structure of the ACE measurement is not validated and different among Black SGMs compared to other intersectional groups, inequities observed for Black SGMs might be an artificial result due to differences in measurement performance rather than true differences. Findings using flawed psychometric measures may make erroneous conclusions about inequities, it may fail to highlight existing inequities or identify new inequities when there were none [11]. To our knowledge, no studies have examined the item-by-item performance of the measure among any population, and even fewer examined ACEs specifically among Black SGMs.

Furthermore, past studies using population-based data from the BRFSS usually only have a small number of Black SGM participants, limiting the potential generalizability and inference [9]. These surveys typically used a single item gender identity question, which is deemed inadequate to identify transgender individuals [12]. Past studies cannot observe potential inequities within more granular intersections, such as Black SMM from lower socioeconomic status. Moreover, measurement of sexual orientation in population studies are often susceptible to misclassification biases, as some SMMs (particularly from racially minoritized groups) are unwilling to disclose their orientation in a government survey [9, 13]. Hence, the purpose of the current study is to examine the psychometric properties of the BRFSS ACE measure from an existing cohort of Black SMMs and gender expansive communities in Southern Chicago through factor analysis and Item Response Theory [14, 15].

## Methods

Data were taken from the Neighborhood and Networks (N2) cohort study, an ongoing longitudinal study that recruited individuals that were assigned male at birth (including SMMs, transgender woman and non-binary individuals) in Chicago. Convenience sampling from a community health center and peer referral were used to recruit participants from February 2018 and onwards. Additional information about study design, including recruitment can be found elsewhere [14, 15]. The present analysis uses the second wave of data from 147 participants not living with HIV from the Chicago site where the ACE question was included. The second wave of N2 data was collected from July 2018 to August 2020. Eligibility criteria at baseline for participants included: 1) reporting age between 16–34 years, 2) identifying as African American or Black, 3) being assigned male sex at birth, 4) reporting a sexual encounter with a cisgender man or a transgender woman in the past year, 5) residing in the Chicago metropolitan area, 6) not planning to move outside of the Chicago MSA area for the next 2 years. Informed consents were obtained verbally during study visits.

### Measures

#### Adverse childhood experiences

ACEs were measured using the BRFSS measure, which had been shown to validated across many populations, including by sexual orientation and race, but not explicitly using an intersectionality framework [8, 9]. The ACEs measure has been shown to have a three-factor structure although summing all the scores up into a single unidimensional measure is also a recommended common practice [8]. The three-factor structure includes components of “Household Dysfunction”, “Emotional / Physical”, and “Sexual Abuse”. There were eight different domains of ACE listed in this scale (see Table 1) which were composed of 11 total items. Participants can answer *yes / no / don’t know* for each item. Less than 10 participants for certain items endorsed the *don’t know* option, to maximize sample size, we coded these responses as *no*. We calculated a total ACE score as well as dichotomizing it into two categories. Experiencing four or more ACE domains was used as a cut-off, as there is a 4 to 12 fold risk of increase in all negative health outcomes for them compared to those with no ACE at all [6, 16].

**Table 1:** Frequencies of the Adverse Childhood Experiences Items Among Black Sexual Minority Men, The N2 Cohort Study (*N* = 147)

Item		N (%)—Domain	Cronbach Alpha if Domain is Removed	N (%)—Item	Cronbach Alpha if Item is Removed	Cronbach Alpha
	Before you were 18 years old,					
	**Factor 1: Household dysfunction**					0.79
	Domain 1: Household Mental Illness (item #1)	27 (18.4)	0.82			
1	did you live with anyone who was depressed, mentally ill, or suicidal? (yes / no / not sure)^a^			27 (18.4)	0.89	
	Domain 2: Household Substance Abuse (item #2 or 3)	54 (36.7)	0.83			
2	did you live with anyone who was a problem drinker or alcoholic? yes / no / not sure)^a^			41 (27.9)	0.89	
3	did you live with anyone who used illegal street drugs or who abused prescription medications? (yes / no / not sure)^a^			38 (25.9)	0.88	
	Domain 3: Incarcerated Family Member (item #4)	56 (38.1)	0.83			
4	did you live with anyone who served time or was sentenced to serve time in a prison, jail, or other correctional facility? (yes / no / not sure)^a^			56 (38.1)	0.89	
	Domain 4: Parental Separation (item #5)	46 (31.3)	0.88			
5	were your parents separated or divorced? (yes / no / not sure / parents never married)^b^			46 (31.3)	0.91	
	**Factor 2: Emotional / Physical Abuse**					0.87
	Domain 5: Intimate Partner Violence (item #6)	49 (33.3)	0.83			
6	how often did your parents or adults in your home: slap, hit, kick, punch, or beat each other up? (scale: never/ once / more than once / not sure)^c^			49 (33.3)	0.90	
	Domain 6: Physical Abuse (item #7)	40 (27.2)	0.83			
7	how often did your parents or adults in your home: hit, beat, kick, or physically hurt you in any way? Do not include spanking. (scale: never/ once / more than once / not sure) ^c^			40 (27.2)	0.90	
	Domain 7: Emotional Abuse (item #8)	79 (53.7)	0.82			
8	how often did your parents or adults in your home: swear at you, insult you, or put you down? (scale: never/ once / more than once / not sure)^c^			79 (53.7)	0.88	
	**Factor 3: Sexual Abuse**					0.98
	Domain 8: Sexual Abuse (any item # 9, 10, 11)	46 (31.3)	0.83			
9	how often did anyone at least 5 years older than you: touch you sexually? (scale: never/ once / more than once / not sure)^c^			44 (29.9)	0.88	
10	how often did anyone at least 5 years older than you: try to make you touch them sexually? (scale: never/ once / more than once / not sure)^c^			41 (27.9)	0.89	
11	how often did anyone at least 5 years older than you: force you to have sex? (scale: never/ once / more than once / not sure)^c^			24 (16.3)	0.89	
	Total ACE score items – mean (SD) – total 11 items – unidimensional structure					0.90
	0			32 (21.8)		
	1			21 (14.3)		
	2			14 (9.5)		
	3			19 (12.9)		
	4 +			61 (41.5)		
	Total ACE score domains – mean (SD) – total 8 items—unidimensional structure					0.85
	0	32 (21.8)				
	1	21 (14.3)				
	2	20 (13.6)				
	3	23 (15.6)				
	4 +	51 (34.6)				

^a^All “don’t know” and “not sure” were classified as no

^b^Those whose parents were “never married” were classified as no

^c^ “Once” and “more than once” were considered as “yes” (given as a score of 1). “Not sure” were considered as no. These scoring systems were consistent with previous publications

#### Depressive Symptoms

This was measured using the Center for Epidemiologic Studies Depression Scale (CES-D 8), an eight-item questionnaire used to measure depressive symptoms which have been validated in many populations, including Black population, SMMs, and TWs [17–19]. The score ranges from 0 to 24, and a score of nine or higher is considered to have probable depression [20].

#### Anxiety

This was measured using the Generalized Anxiety Disorder (GAD-7) scale, which also has been validated in the general population [21]. The score ranges from 0 to 21, and a score of eight or higher is considered to have potential anxiety disorder [21, 22].

#### Housing instability

This was measured using two questions: “Have you been homeless in the past 6 months” and “Are you worried that in the next 6 months you may not have stable housing that you own, rent, or stay in as part of a household?”. Any positive answers to the questions above will be considered to have unstable housing.

#### Socio-demographics

Socio-demographic of participants were also collected, which include gender identity, sexual orientation, age education, income, and employment.

### Statistical analysis

Confirmatory factor analysis (CFA) was first conducted for both the unidimensional and three-factor structure of the BRFSS ACE to confirm the structure of this scale in this sample. The current convention calculated the sum of the eight ACE domains, giving a range of 0–8 instead of summing up the 11 items (see Table 1) [7]. To assess whether the practice of summing up items as a unidimensional construct is also valid, we examined the Cronbach alpha of the 11 items and 8 domains in the ACE measure; a value larger than 0.80 for all the items in the scale means the scale has a reliable structure. This was consistent with the previous validation of the BRFSS ACE measure, where a unidimensional interpretation of ACE was considered to be equally robust when the overall Cronbach was still high [8].

Comparative fit index (CFI), Tucker Lewis Index (TLI), and root mean square error of approximation (RMSEA) were used to assess the fit of the CFA, with the commonly accepted cut-off as an indication of good fit (CFI, TLI > 0.95, and RMSEA < 0.06). [23]. As there are no gold standard measure of ACEs (i.e., ACEs measurements were self-reported retrospectively as an adult), we cannot assess its psychometric validity. However, we examined concurrent validity by examining how the BRFSS measure is associated with depressive symptoms and anxiety in adulthood, which are known to be strongly associated with ACE [1, 3]. As we wished to assess the overall range of ACE scores by assessing concurrent validity, we first used a Pearson correlation coefficient to assess the strength of association between ACE and full depressive symptoms and anxiety scores. We also presented a linear regression result between ACE scores with depressive symptoms and anxiety scores while adjusted for all demographic factors. To fully understand concurrent validity, we also calculated the risk ratio of having probable depression and anxiety disorder from experiencing ACE using a modified Poisson regression [24]. Due to the small sample sizes, we cannot assess measurement and configural invariance by demographic characteristics, but we examined demographic differences in ACE scores to consider any intersectional differences between them [2, 7, 25].

We also ran a 2-parameter Item Response Theory (IRT) model for dichotomous traits, which measures a latent trait for each item as a function of item severity and discrimination [26–28]. Severity is defined as the point where there is a 50% chance that the item is present. Note that severity is inversely proportional to the item prevalence, the rarer the item is, the more likely that participants who endorsed that item has severe ACE. On the other hand, discrimination is the ability for each item to differentiate participants with latent trait levels above or below the item severity. Discrimination is represented as the slope in the Item Characteristics Curve (ICC) [28]. The point on the x-axis of the ICC where the y-axis is 0.5 represents the severity of the item.

The item-by-item fit were assessed using Orlando and Thissen criteria of RMSEA X^2^ for dichotomous items, as well as with infit and outfit mean square statistics [27, 29]. The person level fit was assessed by the Z-standardized infit and outfit statistics [30]. The X^2^ statistic is based on the observed residuals, a good fit would mean we do not reject the null that the observed value for the item fit the expected value from the model; RMSEA X^2^value of lower than 0.05 indicates a good fit. The infit and outfit statistics can be measured at an individual and item level [30]. The outfit statistics is outlier-sensitive, it is sensitive to extreme departures from model expectations (i.e., when it gives a probability of close to 0 or 1); for example, at the individual level, it will identify a person with a high ACE severity who do not endorse an ACE item that is typically endorsed by everyone else with high ACE. On the other hand, infit statistics is inlier-sensitive, meaning that it is sensitive to less extreme departures from the model expectations (i.e. along the middle of the probability continuum), for example, a person with low ACE severity endorsed an item that is less common for someone in that group to endorse. Ideally, the infit and outfit statistics for items should be between 0.5 and 1.5 [29, 31]. For both statistics, being higher than this range is a larger concern as it is an indication that the item is not fitting well, whereas being lower than 0.5 might be an indication of overfitting [30, 31]. For the person fit, the Z-standardized infit and outfit should be between -1.96 and 1.96 [30, 31].

To ensure robustness of results, we also reran all analyses where participants with any “don’t know” responses were excluded (n = 99). We used R 4.3.1 using the psych, lavaan and mirt packages, to conduct all analyses [32–34].

## Results

All 147 participants in the N2 cohort were assigned male at birth. Participants’ age ranges from 16 – 34, with a mean age of close to 25, with 63% being 25 years old or older. Of participants, 85% identified as male, 10% as transgender and 5% as non-binary or another gender expansive identity. Of participants, 53% identified as gay, 35% as bisexual and over 11% as another category. The cohort came from a low socioeconomic background, 44% of the cohort were housing insecure, about 40% made than $10,000 or less annually. About 42% of participants reported having paid employment in the past year. Finally, 40% of participants reported having a high school diploma or equivalent; 25% did not finish college and about 10% did not finish high school. About 20% never had any ACEs, while 34% had endorsed 4 or more ACE domains. In the current sample, the most common ACE domains is emotional abuse (53%), followed by incarcerated family members (38%) (see Table 2).

**Table 2:** Sociodemographic Characteristics of Black Sexual Minority Men in the N2 Study living in Chicago and completed the adverse childhood experiences questions

	n (%)
Age—mean (SD) (Range: 16 – 34)	24.82 (3.84)
Age categories	
16—24	54 (36.7)
25 +	93 (63.3)
Gender Identity	
Male	125 (85.0)
Transgender female or Trans woman	15 (10.2)
Non-binary or another identity	7 (4.8)
Sexual Orientation	
Bisexual	50 (34.5)
Gay	78 (53.8)
Another sexual identity	17 (11.7)
Housing Instability	62 (44.3)
Having any paid employment	69 (42.9)
Past year income in USD (%)	
< 5,000	35 (23.8)
5,000—9,999	25 (17.0)
10,000—19,999	41 (27.9)
20,000—29,999	20 (13.6)
30,000—39,999	18 (12.2)
40,000 +	8 ( 5.4)
Education (%)	
Less than high school diploma	20 (13.6)
High school diploma or equivalent	56 (38.1)
Some college, no degree	37 (25.2)
Associate degree	19 (12.9)
Bachelor's degree	13 ( 8.8)
Post graduate degree	2 ( 1.4)

## Factor analysis

The three-factor structure CFA solution fits the data well, with CFI = 0.975, TLI = 0.967, and RMSEA = 0.051, all meeting the recommended cut-offs [23]. All the three subscales also have good reliability measures. The measures “Emotional / Physical abuse”, “Household Dysfunction”, and “Sexual Abuse” have Cronbach’s alpha of 0.79, 0.87, and 0.98 respectively (see Table 1 for details). The fits from the CFA with unidimensional models from 11 items and 8 domains were not as acceptable as the one from the three-factor structure. However, the unidimensional scale still has a high reliability with Cronbach alpha of 0.90 (95% CI: 0.78, 0.97) from the 11-item model, and a Cronbach alpha of 0.85 (95% CI: 0.62, 0.97) with the 8-item model, suggesting that while the three-factor model fit the data best, these items may belong to one higher order latent class of ACE.

## Concurrent validity

We examined concurrent validity by first testing whether the average ACE differed by gender identity, sexual orientation, age groups, employment status, income, education levels, and housing instability. There were no statistically significant differences between the sociodemographic groups in the average ACE scores using ANOVA or t-test (see Table 3). Similarly, when we compared the proportion of those experiencing 4 or more ACEs between these sociodemographic groups using a chi-square test, there were no statistically significant association that emerged (data not shown). Regardless of statistical significance, certain groups appeared to have higher ACE scores relative to others. Those with income < $5,000, those who were transgender, non-binary, or having another identity and those with less than a high school degree appeared had the highest ACEs compared to other groups within each respective sociodemographic category.

**Table 3:** Comparing Adverse Childhood Experience Scores between different sociodemographic groups of Black Sexual Minority Men in the N2 Study

*N* = 147	Categories (n)	Mean ACE score (0 – 8)	*p*-value
Sexual orientation	Bisexual (*n* = 50)	2.72	0.891
	Gay (*n* = 78)	2.74	
	Another identity (*n* = 19)	2.47	
Gender Identity	Cis male (*n* = 125)	2.65	0.493
	Transgender, non-binary or other (*n* = 22)	3	
Income	< $ 5,000 (*n* = 35)	3.31	0.25
	$ 5,000 – 9,999 (*n* = 25)	1.88	
	$ 10,000 – 19,999 (*n* = 41)	2.83	
	$ 20,000 – 29,999 (*n* = 20)	2.6	
	$ 30,000 – 39,999 (*n* = 18)	2.44	
	$ 40,000 +	2.75	
Education	Less than high school (*n* = 20)	3.6	0.174
	High school diploma or equivalent (*n* = 56)	2.45	
	Some college, no degree (*n* = 37)	2.27	
	Associate degree (*n* = 19)	3.05	
	Bachelor’s degree or higher (*n* = 15)	3.07	
Housing Instability	Unstable Housing (*n* = 62)	2.95	0.248
	Stable Housing (*n* = 85)	2.52	
Paid Employment (is this past 2 weeks?)	Employed (*n* = 69)	2.84	0.467
	Unemployed (*n* = 78)	2.58	
Age categories	16 – 24 (*n* = 54)	2.83	0.591
	25 + (*n* = 93)	2.62	

There were significant correlations between ACE scores with depressive symptoms and anxiety scores, both prior and after covariate adjustments. The overall Pearson correlation between the total ACE domains and depressive symptoms was 0.246 (95% CI: 0.088, 0.39), while between ACE and anxiety was 0.26 (95% CI: 0.10, 0.40). Adjusted for all other sociodemographic characteristics (age, sexual orientation, gender identity, income, education, employment, and housing instability), for every additional increase in one ACE domain an individual experienced, depressive symptoms scores increased by 0.484 points (95% CI: 0.128, 0.841); for every increase in one ACE domain, anxiety scores increased by 0.592 points (95% CI: 0.190, 0.995). There was also a significant correlation with probable depression, adjusting for the same sociodemographic factors above, for every additional increase in one ACE domain an individual experienced, the risk of probable depression increased by 16% (Risk Ratio [RR]: 1.16, 95% CI: 1.01, 1.34. The relationship remained significant when comparing those with 4 or more ACE domains, where risk of probable depression is twice more likely than those with fewer than 4 ACEs (RR: 2.30; 95% CI: 1.25, 4.22) after adjusting for sociodemographic factors. There was no statistically significant increase in risk of developing anxiety, although the risk ratios were still above 1 (see Table 4). There were also some statistically significant correlations when we scored ACE by items rather than domains (i.e. summing 11 items instead of 8; see Appendix 1), however these were strongest when summing all 8 domains.

**Table 4:** Association between depressive symptoms, anxiety, and Adverse Childhood Experiences within the N2 Study of Black Sexual Minority Men (*n* = 147)

	Unadjusted Results		Adjusted Results^a^	
	Depressive Symptoms (scores: 0 – 24)	Anxiety (scores: 0 – 21)	Depressive Symptoms (scores: 0 – 24)	Anxiety (scores: 0 – 21)
	Beta coefficient (95% CI)		Beta coefficient (95% CI)	
ACE (scores: 0–8)	0.514 (0.181, 0.845)	0.604 (0.231, 0.977)	0.484 (0.128, 0.841)	0.592 (0.190, 0.995)
ACE: 4 + domains vs < 4 domains	1.960 (0.408, 3.511)	2.556 (0.820, 4.293)	1.964 (0.296, 3.632)	2.559 (0.679, 4.440)
	Probable Depression vs No depression: Risk ratio	Anxiety vs No anxiety: Risk ratio	Probable Depression vs No depression: Risk ratio	Anxiety vs No anxiety: Risk ratio
ACE (scores: 0–8)	1.18 (1.03, 1.35)	1.07 (0.95, 1.22)	1.16 (1.01, 1.34)	1.08 (0.94, 1.26)
ACE: 4 + domains vs < 4 domains	2.15 (1.14, 4.05)	1.25 (0.70, 2.25)	2.30 (1.25, 4.22)	1.28 (0.68, 2.41)

^a^Adjusted for all sociodemographics listed in Table 3 (age, sexual orientation, gender identity, income, education, employment, and housing instability)

## Item response theory (IRT)

Given the high Cronbach alpha for the unidimensional measure and summing up all the eight domains of the BRFSS ACE is the current convention when analyzing this measure, we proceeded with running a 2 parameter models for IRT using the 8 domains of ACE.

No items showed significant item fits using the RMSEA X^2^ statistic; all RMSEA X2 were less than or close to 0.06, suggesting that there were no major concerns with the item fits (Table 5) [27, 29]. The domains showing less than ideal fits were sexual abuse and emotional abuse, which were both between 0.06 – 0.07 [23]. These were still evidence of good fit, although the lower RMSEA might also be due to the fact that they are part of the other subscales in the three-factor structure (Table 1).

**Table 5:** Severity and Discrimination Indices of the Eight Adverse Childhood Experience Domains Found in the N2 Study

Domain		Discrimination (Slope Parameters)	Severity	RMSEA X2	Outfit	Infit
1	Household Mental Illness	2.092	1.169	0.00	0.526	0.877
2	Household Substance Abuse	1.687	0.492	0.00	0.702	0.876
3	Incarcerated Family Member	1.394	0.484	0.00	0.785	0.900
4	Parental Separation	0.385	2.113	0.039	0.983	0.987
5	Intimate Partner Violence	1.842	0.598	0.025	0.644	0.869
6	Physical Abuse	1.921	0.821	0.00	0.624	0.869
7	Emotional Abuse	2.672	-0.091	0.064	0.475	0.687
8	Sexual Abuse	1.537	0.733	0.069	0.724	0.906

**Fig. 1:**
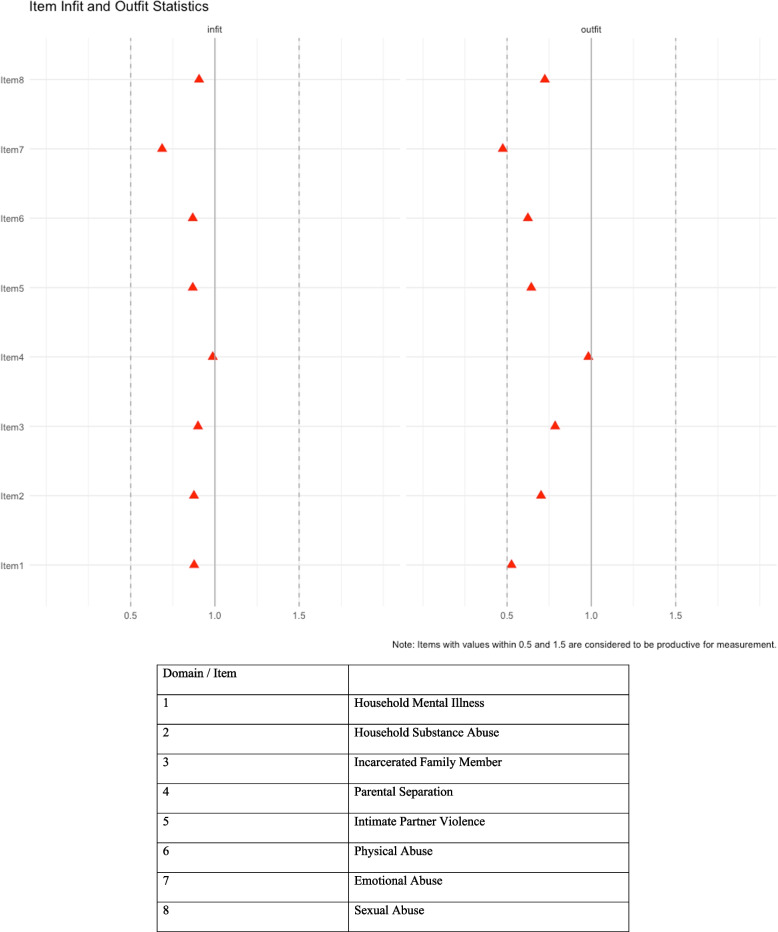
Infit and Outfit Statistics of the two-parameter Item Response Theory Model on Black SMM in the N2 study

Observing the infit and outfit statistics, most items appeared within the range of 0.5 and 1.5, suggesting that there were no issues with the item fits (Fig. 1) [35]. No items have infits outside of the recommended range. When assessing the person fit, there were only two individuals (< 5%) who had Z-standardized infit and outfit values outside of the recommended -1.96 to 1.96 range, which indicates no concern (see Appendix 2) [29].

**Fig. 2:**
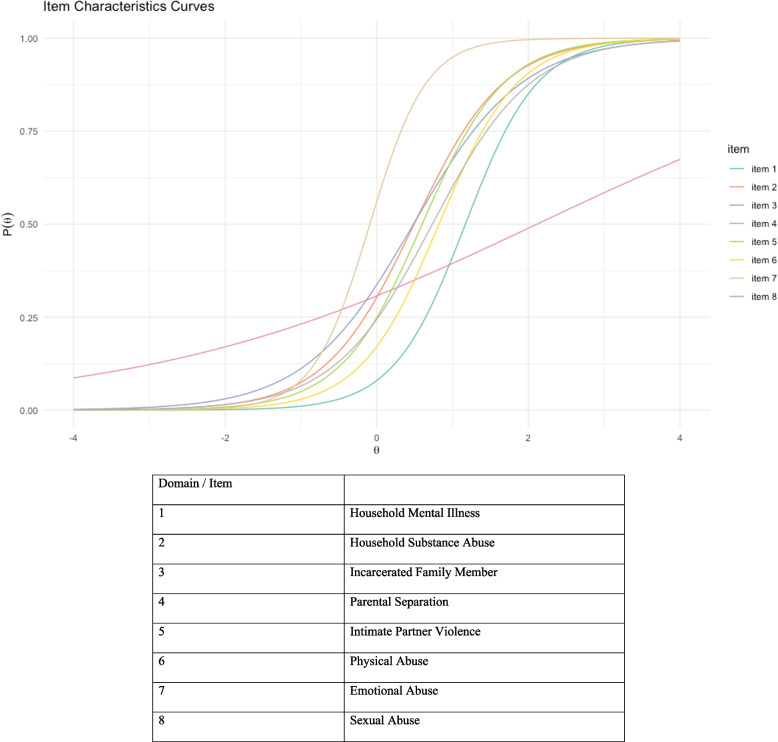
Item Characteristic Curves for the Eight Domains of Adverse Childhood Experiences in the N2 study

The discrimination and severity indices were illustrated in the ICC curves in Fig. 2 and Table 5. These results suggest that most items had acceptable severity and discrimination index. The slope parameters (discrimination index) ranged from 0.385 to 2.672. As can be seen in the ICC curves, the item that had the poorest performance is parental separation, the slope for this item is the lowest relative to every other item. This item does not appear to discriminate between those who have high ACE and low ACE [36]. Emotional abuse appeared to be best at discriminating between those with and without ACE, as it has the highest slope of 2.677, but it has the lowest severity parameter (-0.091). This means that while emotional abuse may distinguish between those with high ACE and low ACE, it is not a sign of higher ACE severity.

For all the above analyses, we ran sensitivity analyses where any participants with “don’t know” responses were removed (*n* = 99), it did not alter any conclusions from the main analysis.

## Discussion

We conducted a factor analysis and item response theory analysis on the commonly used BRFFS measure of ACE among a well-characterized sample of Black SMM and transgender women in Southern Chicago with high rates of unstable housing and unemployment. Their rates of ACEs were extremely high, where about 34% have had 4 or more ACEs. In contrast, previous report using the BRFSS data found that 13.3% of United States adults have had 4 or more ACEs, while 34% had experiences 0 ACEs [7]. The CFA supported the known three-factor structure of ACE well. A unidimensional structure with the 8 domains of ACE being summed up was also appropriate as it showed acceptable reliability and was most correlated with scores depressive symptoms and anxiety, as well probable depression as an adult. Unlike previous findings with other populations, the domain “parental separation” fits the hypothesized structure poorly and the discrimination and severity indices were poor compared to other items, suggesting that it is unable to distinguish those experiencing high and low ACEs.

Many studies used the BRFFS ACE measure to compare the effects of ACE between different populations such as by sexual orientation, race, or gender [7, 9]. We have identified a highly marginalized cohort of Black SMMs and TW, and our findings have implications for health equity research. If a study compared two different population with respect to their ACE levels, it is unknown if results were driven due to one group not endorsing the parental separation item the same way as the other group in the study. About 30% of the participants’ parents in the current study were never married, which may also indicate this item does not work well in this population (see Appendix 3). Furthermore, it is worth noting that this is a younger group of Black SMM and TW (ages 16 – 34), majority are United States citizens, which means their parents also grew up in era where marriage in the Black community were even less common [37]. This finding supports previous studies that found underserved Black communities may require an alternative measure such as the expanded ACE (ACE-E) to fully capture the cumulative negative effects of ACE and experiences of community adversity [5].

While measurement invariance has been established among Black communities and by sexual orientation, no studies to our knowledge has performed an IRT on this ACE measure or more broadly assessed measurement invariance across intersectional positions within a sample of Black SMM, transgender women, and other gender expansive persons. This is the first indication that further examination of this ACE measurement is required, possibly in other populations where there might be higher rates of parents who were not married or separated. Even though the ACE measure has often demonstrated the ability to hold measurement invariance across populations, it does not mean all items belong to the scale.

Future health equity research may want to run sensitivity analysis with the particular parental separation item, especially when examining Black SMMs, transgender women, other gender expansive categories, and other marginalized groups. More studies should also be done with IRT on different populations with this measurement to ensure it is performing as expected. With the changing socio-demographic structure worldwide, declining marriage rates, and increasing divorce rates, it might be worthwhile re-examining the utility of this item in an ACE measure. If the intention of the item is to measure household stability during childhood, this may need further re-examination, especially for SMMs and TW, who may have support from a “chosen family” instead of biological family [38, 39]. More studies should also consider the idea of “counter ACE”, which is a newer term that considers how positive childhood experiences (such as having emotional support) may buffer the negative consequences of experiencing ACEs [40, 41]. This may further elucidate the impact of ACE on mental health, particularly among marginalized populations.

A better understanding of ACE measurements will help identify the ACE components that contribute to any health inequities (or lack thereof) and help direct policies that can improve childhood experiences and reduce inequities in ACEs. The effect of different components of ACEs might differ between communities, especially in groups that experienced community adversities [5]. One ACE component that is salient in one group (e.g. higher incarceration) may not be salient in another group. Thus, improvements on these measurements will not only benefit Black SMMs and TW, but also other intersectional group in which ACE can have a substantial impact. The Center for Disease Control and Prevention (CDC) listed potential strategies and policies (such as family friendly work policies, economic support for families) that can be enacted to reduce violence and adversity for children [42, 43]. However, more implementation research is required, as the social, environmental, political, and economic context of these policies cannot be overlooked when studying ACEs [42, 43]. While the current study cannot infer the effectiveness of these policies, future studies should also be conducted to assess whether alternatives to incarceration and creating stronger family units will have downstream impact on reducing ACE experiences among everyone, especially among those who are most marginalized. In particular, policies should critically consider the context and situations of Black families whenever assessing strategies to address ACE in order to prevent any actionable policies that may inadvertently perpetuate harm to the community.

This is the first study that examined ACE among Black SMM and Black transgender women to our knowledge. Often the number of participants from large population surveys that falls under this particular intersection is too small and even in our study, we were unable to disaggregate by gender identity, due to the sample size. For example, we were unable to run smaller intersectional analysis (e.g., Black TW vs cisgender Black SMM), but this is the first step to understanding ACE among Black SMMs and Black transgender women. While the current study is still limited in sample size, it allows us to examine a group of highly disadvantaged Black SMMs and TWs, which would be difficult to do from large population surveys in the BRFFS. Although there were no statistically significant differences in ACE scores between these intersectional groups, those who made < $5,000, less than high school degree and transgender appeared to have the highest ACE scores compared to all other groups. Our findings of statistical non-significance might be driven by sample size. Furthermore, while our recruitment strategy has enabled us to recruit some of the most marginalized communities of Black SMMs and TWs (i.e., younger Black SMM and TWs living in predominantly Black neighborhoods with lower socioeconomic status and high unemployment), this also means the results may not be generalizable to all Black SMM and TW communities. Our results should also not be used to make inferences about the prevalence of ACE in this community. It is unknown if the true prevalence of ACE in the community is as high as we found, or if it is an artifact of the sample. Finally, there might be some measurement errors from the self-reported mental health measures (e.g. depressive symptoms, anxiety), as well as errors from a retrospective measure of ACE that asked participants to recall traumatic experiences from childhood [44].

## Conclusions

The BRFFS ACE is appropriate to be used among Black SMM and TW with the commonly accepted use of summing up all 8 domains. The 3-factor structure in this population is also confirmed. However, there is a concern that the parental separation domain may not be a good fit in this population. Further IRT analyses with the BRFFS ACE measure might be warranted with other populations to foster equitable research for all populations, not just among Black SGMs. As there are many large datasets with BRFFS ACE measures beyond Black SMM [6, 7, 9], IRT can be done with these items on larger sample sizes of BRFFS data. Given this, caution may need to be taken when computing analysis comparing Black SGMs with other populations for health equity research, as results might be driven spuriously by parental separation which does not belong for this population. We recommend running sensitivity analyses by excluding this item when comparing this population of Black SGM to other groups to ensure robust results.

### Supplementary Information

**Supplementary Material 1. **

## Data Availability

No datasets were generated or analysed during the current study.

## Abbreviations

ACE: Adverse childhood experience
IRT: Item Response Theory
CFA: Confirmatory Factor Analysis
CFI: Comparative fit index
RMSEA: Root Mean Square Error of Approximation
TLI: Tucker Lewis Index
SMM: Sexually Minoritized Men
BRFSS: Behavioural Risk Factor Surveillance System
ICC: Item Characteristics Curves
CES-D: Center for Epidemiologic Studies Depression Scale
TW: Transgender women
SGM: Sexual and gender minorities
CDC: Center for Disease Control and Prevention
