# Supplementary material for: Adverse childhood experiences among black sexually minoritized men and Black transgender women in Chicago
Source: Int J Equity Health. 2024 Apr 16;23:74. doi: 10.1186/s12939-024-02168-6 (PMC11020455; doi:10.1186/s12939-024-02168-6)
Supplement: Supplementary file 1 — Supplementary Material 1. [file 12939_2024_2168_MOESM1_ESM.docx]

Appendix 1. Association between the sum of all 11 ACE Items with Depressive Symptoms and Anxiety

|  | Unadjusted Results | | Adjusted Results* | |
| --- | --- | --- | --- | --- |
|  | Depressive Symptoms (scores: 0 – 24) | Anxiety (scores: 0 – 21) | Depressive Symptoms (scores: 0 – 24) | Anxiety (scores: 0 – 21) |
|  | Beta coefficient (95% CI) | | Beta coefficient (95% CI) | |
| ACE (scores: 0-11) | 0.356 (0.103, 0.610) | 0.435 (0.150, 0.719) | 0.353 (0.082, 0.625) | 0.435 (0.128, 0.742) |
|  | Probable Depression vs No depression:  Risk ratio | Anxiety vs No anxiety:  Risk ratio | Probable Depression vs No depression:  Risk ratio | Anxiety vs No anxiety:  Risk ratio |
| ACE (scores: 0-11) | 1.060 (0.991, 1.245) | 1.057 (0.946, 1.177) | 1.064 (0.982, 1.252) | 1.063 (0.942, 1.198) |

*adjusted for all sociodemographics listed in Table 3 (age, sexual orientation, gender identity, income, education, employment, and housing instability).

Appendix 2. Infit and Outfit Statistics of Individual response


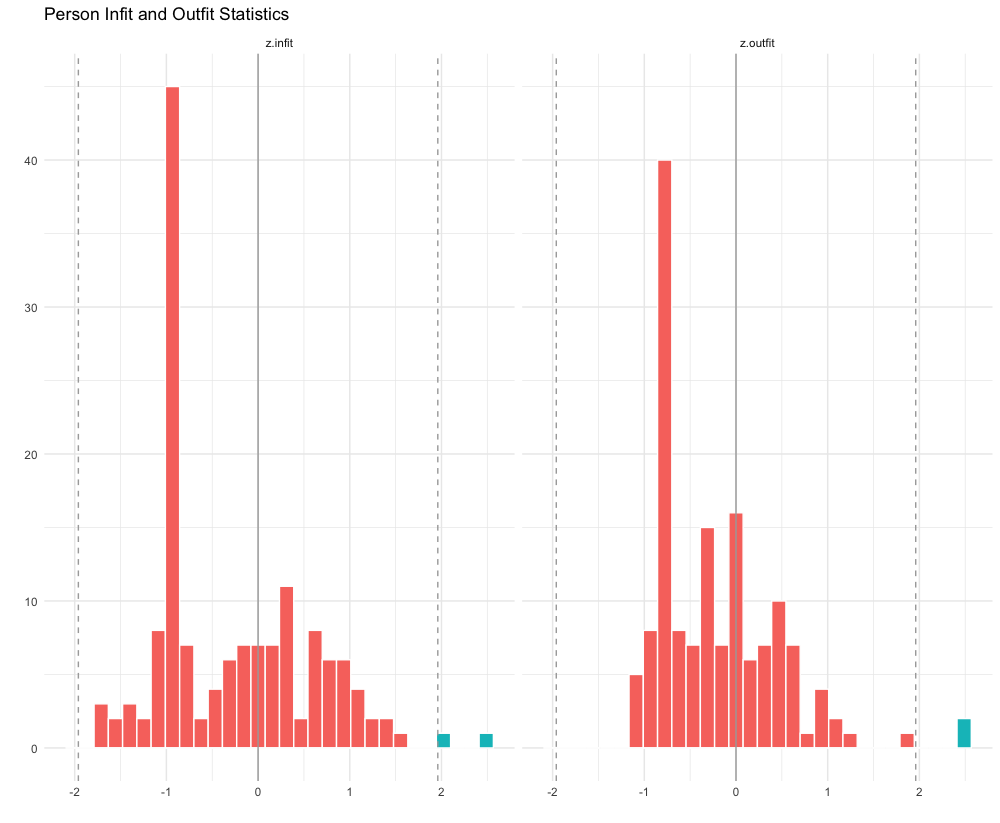


Appendix 3. Confirmatory Factor Analysis and Item Response Theory Results when Participants with Parents who were never married were removed (n = 97)

3a. Confirmatory Factor Analysis results:

|  | Unidimensional 1 factor structure result | 3 factor structure results (with loadings as specified in Table 1) |
| --- | --- | --- |
| RMSEA | 0.144 (0.103, 0.187) | 0.075 (0.034, 0.110) |
| CFI | 0.795 | 0.949 |
| TLI | 0.713 | 0.931 |

Fit statistics does not fit the model as well possibly due to reduced sample size

3b. Item Response Theory Results:

| Domain |  | Discrimination (Slope Parameters) | Severity | Outfit | Infit |
| --- | --- | --- | --- | --- | --- |
| 1 | Household Mental Illness | 2.931 | 1.034 | 0.369 | 0.861 |
| 2 | Household Substance Abuse | 1.750 | 0.661 | 0.706 | 0.930 |
| 3 | Incarcerated Family Member | 1.353 | 0.530 | 0.809 | 0.935 |
| 4 | Divorced Family | 0.620 | 0.184 | 0.955 | 0.964 |
| 5 | Intimate Partner Violence | 2.400 | 0.451 | 0.559 | 0.869 |
| 6 | Physical Abuse | 3.160 | 0.678 | 0.383 | 0.803 |
| 7 | Emotional Abuse | 2.602 | 0.096 | 0.518 | 0.790 |
| 8 | Sexual Abuse | 1.715 | 0.791 | 0.712 | 0.938 |


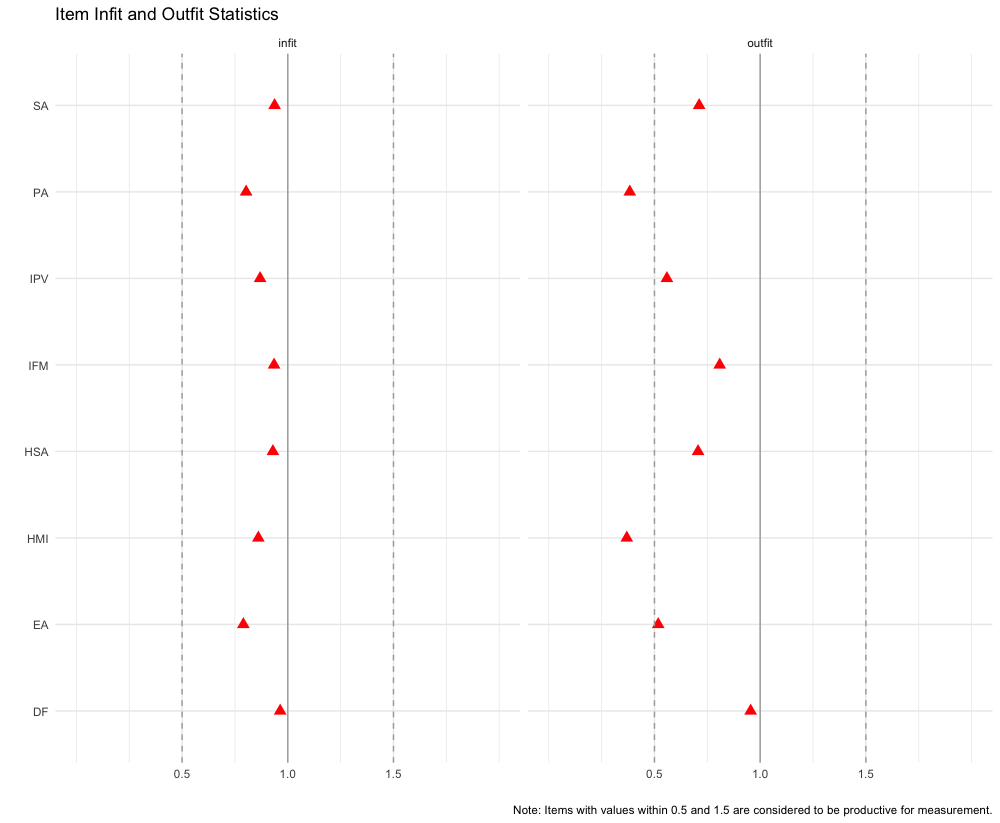


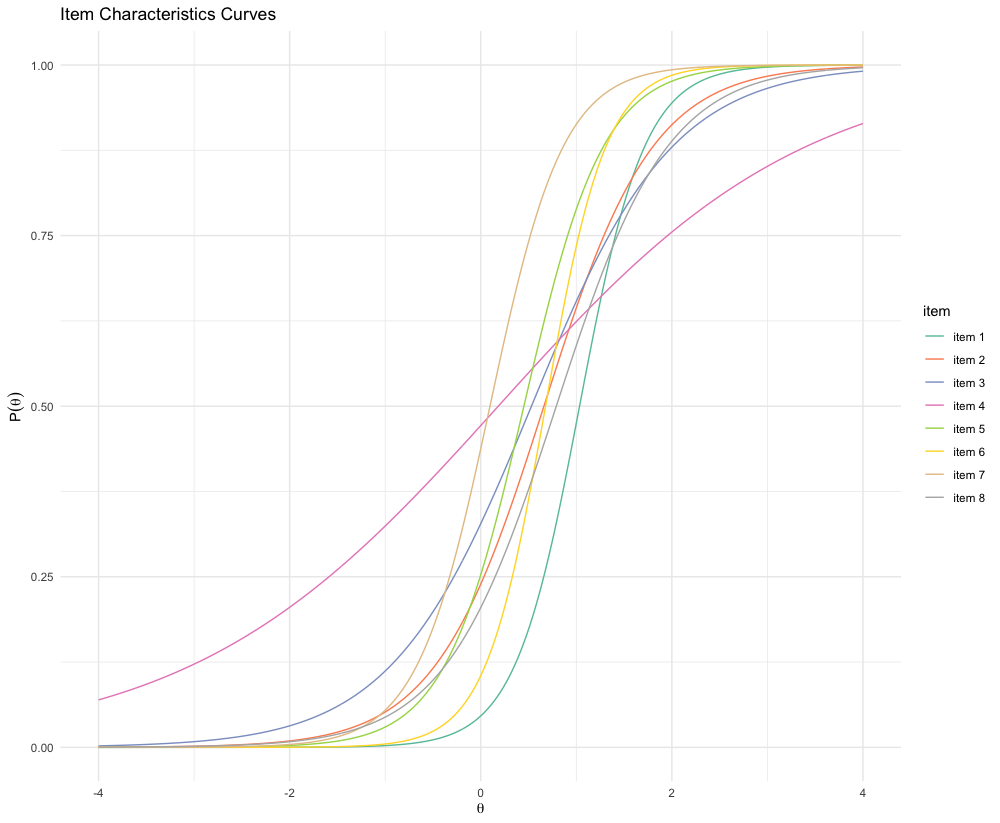


| Domain / Item |  |
| --- | --- |
| 1 | Household Mental Illness |
| 2 | Household Substance Abuse |
| 3 | Incarcerated Family Member |
| 4 | Divorced Family |
| 5 | Intimate Partner Violence |
| 6 | Physical Abuse |
| 7 | Emotional Abuse |
| 8 | Sexual Abuse |
